# Feasibility Study of the Boston Naming Test for the Arab Population

**DOI:** 10.7759/cureus.52351

**Published:** 2024-01-16

**Authors:** Hadeel A Basura, Mohammed Mudarris, Fatimah B Almubarak, Shahad A Alzahrani, Hajar Alghamdi, Ahlam Alsulami, Ameerah Alnakhli, Ghadi Alzahrani, Haythum O Tayeb

**Affiliations:** 1 Faculty of Medicine, King Abdulaziz University, Jeddah, SAU; 2 Health, Medical, and Neuropsychology Unit, Leiden University, Leiden, NLD; 3 Department of Neurology, Neuroscience Research Unit, Faculty of Medicine, King Abdulaziz University, Jeddah, SAU

**Keywords:** bnt arabic version, boston naming test, lexical familiarity, picture naming, neuropsychological tests

## Abstract

Introduction

The Boston Naming Test (BNT) is a widely used US neuropsychological evaluation of confrontation naming for the examination of adults and children with learning disabilities and diagnosis of communication disorders, aphasia, dementia, and acquired brain injury or dysfunction. The purpose of the present study is to evaluate the practicality of the original English version of the 60-item BNT (BNT-60) on an Arab population and the need for a new adaptive Arabic version sensitive to cultural biases and to offer normative data that can serve as a reference for researchers and clinicians in the Gulf region, especially the Kingdom of Saudi Arabia (KSA). Data relating to the familiarity degree of the BNT-60 were also collected.

Methods

This research involved 105 randomly selected and cognitively healthy college students who were native Arabic speakers recruited in Jeddah. The Montreal Cognitive Assessment (MOCA) was administered with a cutoff score of 26. The participants were examined for naming accuracy, naming agreement, and familiarity in using the BNT-60. The data were then analyzed and compared with the findings from studies conducted in the United States.

Results

The BNT-60 was administered to 105 university students from the KSA, and the results were compared with the BNT-60 booklet norms (second edition). Their average performance was noticeably below the norms established by the original test standards. Compared with the participants in the US studies, the participants made approximately 65% more errors on the items including pretzel, wreath, beaver, harmonica, acorn, stilts, harp, hammock, knocker, pelican, muzzle, unicorn, funnel, accordion, asparagus, tripod, yoke, and trellis and 25% more errors on the items including seahorse, dart, igloo, sphinx, palette, and abacus. The item "boomerang" was not compared with the US sample because of differences in the version of the BNT, but the errors in naming this item were as frequent as those in naming the other misrecognized items. The internal consistency among the items’ degrees of familiarity was also very high (α = 0.966), and a significant connection (r = 0.837, P < 0.001) was observed between object familiarity and naming accuracy. The Arabic-speaking population in the KSA and English-speaking population in the United States showed very different levels of familiarity with numerous items.

Conclusion

The participants’ familiarity with the BNT objects varied depending on their culture and impacted their naming accuracy and overall scores on the test. Accordingly, the possibility of cultural biases should be considered when administering the BNT to the population of the KSA and the possibility of making changes so that the test better reflects the Arab culture as suggested.

## Introduction

The Boston Naming Test (BNT) is a popular confrontation naming test used in the United States for the neuropsychological evaluation of adults and children with learning impairments and the diagnosis of communication problems such as aphasia, dementia, and acquired brain injury or malfunction. The first version of the test consisted of 85 items, and subsequent research led to the development of modified versions with 60 and 15 items. The questions are based on black-and-white line drawings of objects ranging with respect to the difficulty of identifying them from common to uncommon. If a correct response is not made within 20 seconds, the examiner may provide a stimulus cue (i.e., a phonemic cue) and then show the participant a list of words on the back of the picture from which the correct one can be chosen [1-4].

In representing the population-specific frequency of the correct answers, numerous researchers have adapted the original items of the test for use with speakers of languages such as Turkish. They adopted 29 new items (5). Portuguese researchers have adapted 20 items (6), and Korean researchers have developed 50 new items (7). Additionally, other English-speaking countries, including Australia and New Zealand, have produced population-specific versions of the BNT and modified two items from the original test (9). The main reason for the development of these other versions is that the test instrument is highly sensitive to the cultural context; hence, it is insufficient simply to translate it into another language [5]. Other factors may also significantly affect BNT scores. For example, there is consistent evidence that educational level is a significant variable in this regard, and several studies have found that naming ability decreases with age, although the influence of gender remains unclear [5-11].

In both the Turkish (5) and Swedish (8) studies, when they adapted their new versions of the BNT-60, they initially conducted a pilot study with a sample size of 120 participants for the former and 111 for the latter. After identifying the items that required adaptation, they proceeded with the development of new suggested items and the norms determination of the study.

Even with the strong evidence of the reliability of the BNT for measuring linguistic abilities, researchers have not yet assessed its feasibility for use in clinical and research settings in the KSA. Therefore, the primary objective of the present study is to assess the applicability of the original BNT for native Arabic-speaking populations. The secondary objective is to determine which items need to be replaced, assess the degree of familiarity (FAM) for each object on the BNT-60, and correlate the accuracy of naming the test pictures with the FAM.

## Materials and methods

Sample group

This study involved 105 healthy college student volunteers enrolled at King Abdulaziz University in the city of Jeddah whose first language was Arabic. The participants were required to meet specific selection criteria, including being a college student between the ages of 18 and 25, being born and raised in Saudi Arabia, obtaining a Montreal Cognitive Assessment (MOCA) score of at least 26, and having no history of neurological, psychiatric, or drug abuse disorders. Every participant provided written informed consent to participate in the research.

Instruments

MOCA

Medical professionals use the MOCA test to assess memory loss and other signs of cognitive impairment and screen for those who are at risk for Alzheimer’s disease, other types of dementia, Parkinson’s disease, brain tumors, and drug addiction. The Arabic version was prepared by Ziad Nasr Alden, a Canadian neurologist notable for creating the Arabic version of the MOCA. The scores on the MOCA range from 0 to 30, and 26 or higher is considered a normal score [12].

BNT

Edith Kaplan, Harold Goodglass, and Sandra Weintraub created the first version of the BNT in 1983 with 85 items [4]. After further study, new versions were prepared, one consisting of 60 items and another of 15. The pictures used in the study are line drawings of objects ranging from common to rare, with the responses to be provided within 20 seconds. When the test is administered to normal individuals, it begins with item number 30 based on the assumption that they are familiar with the previous items. When a participant makes six mistakes, the test is to be discontinued. However, in the present study, no pictures were omitted because the aim was to evaluate the effectiveness of all 60 pictures [4].

Procedure

The Ethics Committee of King Abdulaziz University Hospital approved this study on January 15, 2023 (Reference No. 7-23). No legal barrier prevented the use of either the MOCA or the BNT in research settings. The MOCA has open copyright and was taken from the original website (www.mocatest.org), while the BNT-60 was purchased from www.proedinc.com.

Before the study began, the participants were made aware of its goal and provided their informed consent in writing. The examiners also completed a form to collect the participants’ demographic information. Each participant who met the inclusion criteria was then administered the MOCA as a screening tool before the administration of the BNT-60. The participants who achieved a score of at least 26 on the MOCA were then presented with the pictures for the BNT-60 and assessed for 1) naming agreement (NA) and 2) FAM.

*Naming agreement (NA)* refers to the degree of variation in the words that the participants used to identify the picture. Pictures that evoke the same name from most participants have high NA, and those that elicit a variety of names have low NA. Low NA can be because of either misidentification or the fact that there are numerous correct names for the same object [12]. In this study, we calculated the most frequent words used by the participants as proportions.

*Familiarity (FAM)* relates to the prevalence of an object in the experience of language speakers and can be used to estimate the latency period for each object by having the participants rank the pictures on a five-point scale according to their familiarity with them. Thus, the participants were asked to rate their familiarity with the objects, with five indicating very familiar objects, and one indicating objects that were the least familiar. We instructed them to rate the items rather than the drawings of them [12-14].

We introduced the BNT-60 starting from item 1, without adaptation of the items and in the same test order. The participants were asked to name the objects that were shown to them within 20 seconds. Unlike in the administration of the original test, we provided no cues as we aimed to assess the NA for the test items. After the 20 seconds elapsed or the participants named an item, a familiarity assessment was performed by asking them to rank the objects on a scale of one to five.

For scoring, we assigned a value of one for correct Arabic responses and zero for incorrect responses, English responses, and “don’t know” answers. Table 1 presents the items that the participants answered in English. Table 2 presents the items with more than one correct name in Arabic. For all of the tests, trained sixth-grade medical students examined each participant individually in a single 30-45 minute session held in the classrooms and the library at the university. We then collected all of the answers using a Google questionnaire that the examiners filled out.

**Table 1:** Words answered in English by KSA’s population (n=105).

Item No	Item	No of responses
			2	Tree	1
4	House	1
14	Mushroom	5
16	Wheelchair	2
17	Camel	1
18	Mask	8
20	Bench	2
22	Snail	2
25	Dart	3
27	Globe	1
35	Dominoes	10
39	Hammock	1
41	Pelican	1
42	Stethoscope	1
44	Dog mask instead of muzzle	2
45	Unicorn	48
48	Boomerang	1
52	Tripod	1
58	Palette	16

**Table 2:** Name agreement expressed by percentage of frequency.

Item	Name agreement %						
1. Bed	سرير (99.4)						
								2. Tree	شجرة (99.05)						
3. Pencil	مرسام (87.5)	قلم رصاص (4.7)													
								4. House	بيت (92.3)	منزل (6.6)					
5. Whistle	صافرة/ صفارة/ صفيرة (98.05)														
								6. Scissors	مقص (100)						
7. Comb	مشط (100)														
								8. Flower	وردة (80)	زهرة (18.1)					
9. Saw	منشار (96.19)														
								10. Toothbrush	فرشة / فرشاة (50.47)	فرشاة اسنان/ فرشة اسنان (47.62)					
11. Helicopter	هيلكوبتر (94.29)	طائرة مروحية (1.9)	مروحية (0.95)												
								12. Broom	مكنسة (94.28)	مقشة (0.95)					
13. Octopus	أخطبوط (98.1)														
								14. Mushroom	فطر (88.57)						
15. Hanger	علاقة / عليقة (65.71)	علاقة ملابس (28.57)	معلاق/ معلقة / معلاقة (5.71)												
								16. Wheelchair	كرسي (متحرك/ مقعدين/ ذوي احتياجات خاصة/ عجائز/ متنقل/ مرضى/ اعاقة) (67.59)	عربية/ عربة (22.85)	مقعد متحرك (0.95)				
17. Camel	جمل (96.1)	ناقة (2.8)													
								18. Mask	قناع (87.61)						
19. Pretzel	برتزل (12.3)														
20. Bench	كرسي (حديقة/ خشب/ انتظار) (85.56)	مقعد (10.4)					
								21. Racquet	مضرب (تنس/ كرة) (98.9)						
22. Snail	حلزون (91.4)														
								23. Volcano	بركان (91.4)						
24. Seahorse	حصان بحر (55.2)	فرس بحر (4.7)													
								25. Dart	سهم (59)	سهم رمي (0.95)	نبل (0.95)				
26. Canoe	قارب (92.3)	مركب (1.9)	قارب خشبي (0.9)	زورق (1.9)											
								27. Globe	مجسم كرة ارضية (36.1)	كوكب الارض (5.7)	كرة ارضية (46.6)				
28. Wreath	زينة باب (6.6)	تعليقة باب (0.9)					
								29. Beaver	قندس (28.5)						
30. Harmonica	هارمونيكا (9.5)														
								31. Rhinoceros	وحيد قرن (89.5)						
32. Acorn	بلوط (10.4)														
								33. Igloo	بيت (ثلجي/ جليدي/ الإسكيمو) (45.71)	كوخ (ثلجي/ جليدي) (17.14)					
34. Stilts	No right answers														
35. Dominoes	ضومنة (72.38)						
								36. Cactus	صبار (92.38)						
37. Escalator	درج (كهربائي/ متحرك) (41.91)	سلم (كهربائي/ متحرك) (50.48)													
								38. Harp	هارب (1.9)						
39. Hammock	سرير معلق (6.67)	سرير شبكي (3.8)	سرير متأرجح (0.95)												
								40. Knocker	مطرق باب / مطرقة باب (8.56)	قارعة باب (1.9)	دقاقة/ دقاق باب/ مدق باب/ مدقة (9.53)				
41. Pelican	بجع/بجعة (8.57)														
																42. Stethoscope	سماعة (طبيب\دكتور/ طبية) (66.64)	سماعة قلب (3.80)	سماعة (14.28)				
43. Pyramid	هرم (98.1)																						
								44. Muzzle	قناع (6.67)	قناع كلب (14.29)	كمامة/كمام (3.81)	قفل فم كلب (0.95)	لثام (0.95)		
45. Unicorn	حصان سحري (2.86)	حصان أسطوري (1.91)	حصان وحيد القرن (0.95)	حصان خيالي (0.95)											
								46. Funnel	قمع (34.29)	محقن (0.95)					
																47. Accordion	أوكورديون (2.86)						
48. Noose / boomerang	لعبة مرتدة (0.95)																						
								49. Asparagus	اسبرجس (3.8)	هليون (1.9)					
50. Compass	فرجار (56.19)														
								51. Latch	قفل/قفالة/قفل باب/مقفل باب (79)	زرفيرة/زرفيلة/زرفال /زرفون/زرفان/زرفولة (14.28)	أمان باب (0.95)				
52. Tripod	مثبت / مثبت كاميرا (1.9)	قاعدة / قاعدة تصوير (6.66)	حامل /حامل كاميرا (4.7)												
																53. Scroll	رسالة (62.86)	لفافة / لفيفة (1.91)	معروض (-0.95)	برقية (4.76)	مخطوطة (8.56)	خطاب (3.81)	وثيقة (0.95)
54. Tongs	ملقاط / ملقط (82.86)	كماشة (0.95)	لقاطة (0.95)																				
								55. Sphinx	ابو الهول (45.71)						
56. Yoke	No right answers														
57. Trellis	سقف خشبي (0.95)						
								58. Palette	صحن ألوان (3.81)	لوح ألوان (20)	طبق تلوين (0.95)	خشبة تلوين (3.81)	حاملة ألوان (2.86)	قاعدة ألوان (0.95)	صحن رسم (1.91)
59. Protractor	منقلة (40.95)														
60. Abacus	عداد (9.52)	حاسبة يدوية (3.81)	أداة حساب (2.86)	حاسبة قديمة (0.95)	أداة عد صيني (0.95)		

Statistical analysis

We performed the statistical analyses with IBM SPSS version 21.0. We used the independent sample T-test (chi-square) to analyze the mean between the KSA sample and the original BNT norms. We calculated the mean and standard deviation (SD) for the comparison of the FAM rating in our sample with the US sample. Using Cronbach’s alpha, we calculated the BNT-60 familiarity degree of the KSA sample and evaluated the results for internal consistency. We used Spearman’s rank to calculate the correlation between naming accuracy and familiarity.

## Results

The sample consisted of college students recruited from the city of Jeddah. Their mean age was 21 years (SD = 1.79), their age range was 18-25 years, the mean for the extent of their education was three years of undergraduate studies (SD = 1.38), and 51% were female. Table 3 shows the mean scores and standard deviations for both the current study sample and the original BNT-60 norms [4] for adults. The comparison of the original BNT total scores (means and standard deviations) with the current sample showed a substantial difference (T(124) = 20.146, P < 0.001). An independent T-test indicated a significant difference in the mean scores for the current sample and the original BNT norms. The highest score recorded for the KSA population was 50, and the lowest score was 25.

**Table 3:** Mean and standard deviation by age range from the original BNT record booklet (second edition) and KSA samples. KSA (n = 105), original BNT (n = 21). The BNT mean and SD from the original BNT. Norms = spontaneous correct responses + correct responses after giving stimulus cues, while the Arabic mean and SD are based on spontaneous correct responses only.

	Age range	BNT mean	BNT standard deviation
Original BNT-60 norms for adult	18-39	55.8	3.8
Saudi Arabia sample	18-25	37.5	3.8

Along with comparing the average scores of the two populations, we aimed to identify items that are highly sensitive to cultural bias by comparing the patterns in the errors made by the participants from the KSA with those in the errors by native English speakers in an earlier study of the US population, as shown in Table 4 [15]. A difference in the rate of more than 20% was considered significant. The participants from the KSA made around 65% more errors than the participants in the US study on the items pretzel, wreath, beaver, harmonica, acorn, stilts, harp, hammock, knocker, pelican, muzzle, unicorn, funnel, accordion, asparagus, tripod, yoke, and trellis, as well as 25% more errors on the items seahorse, dart, igloo, sphinx, palette, and abacus.

**Table 4:** Difficulty index represented as a percentage of correctly answered pictures for each BNT item. The items that are highlighted in the table indicate the lowest levels of recognition and familiarity. The item noose was replaced by a boomerang in the new (second edition) that we used in this study. M = mean, SD = standard deviation.

Item	Difficulty index		Familiarity			
	USA	KSA	USA (n =98)		KSA (n = 105)	
	(n = 219)	(n = 105)				
			M	SD	M	SD
1. Bed	100	99.05	4.91	0.36	4.96	0.24
2. Tree	100	99.05	5	0	4.9	0.458
3. Pencil	100	92.38	4.99	0.1	4.9	0.491
4. House	100	99.05	4.98	0.14	4.88	0.57
5. Whistle	99.5	98.1	4.93	0.3	4.56	1.03
6. Scissors	99.5	100	4.95	0.31	4.8	0.76
7. Comb	100	100	4.97	0.18	4.81	0.65
8. Flower	100	98.1	4.9	0.4	4.71	0.77
9. Saw	100	96.19	4.91	0.36	4.46	1.22
10. Toothbrush	100	98.1	4.91	0.35	4.86	0.6
11. Helicopter	99.1	97.14	4.91	0.39	4.63	0.95
12. Broom	100	95.24	4.9	0.36	4.68	0.81
13. Octopus	90	98.1	4.84	0.52	4.35	1.31
14. Mushroom	99.5	88.57	4.82	0.57	4.38	1.3
15. Hanger	100	100	4.89	0.42	4.88	0.57
16. Wheelchair	100	91.43	4.88	0.43	4.61	0.95
17. Camel	99.1	99.05	4.74	0.86	4.54	1.15
18. Mask	98.6	89.52	4.34	1.31	4.41	1.25
19. Pretzel	92.2	12.38	4.83	0.56	3.92	1.52
20. Bench	99.5	96.19	4.88	0.43	4.81	0.66
21. Racquet	100	99.05	4.88	0.42	4.58	1.04
22. Snail	95.4	91.43	4.83	0.54	4.38	1.31
23. Volcano	97.7	96.19	4.72	0.72	4.36	1.35
24. Seahorse	84.9	60	4.42	1.23	3.9	1.6
25. Dart	98.6	60.95	4.57	0.85	4.21	1.24
26. Canoe	100	97.14	4.83	0.56	4.64	0.95
27. Globe	96.8	88.57	4.89	0.37	4.68	0.95
28. Wreath	99.5	7.62	4.76	0.66	3.64	1.59
29. Beaver	97.5	28.57	4.7	0.72	3.74	1.57
30. Harmonica	96.8	9.52	4.38	1.26	3.52	1.65
31. Rhinoceros	90.4	89.52	4.68	0.73	4.36	1.3
32. Acorn	93.6	10.48	4.38	1.2	4.26	1.31
							33. Igloo	99.1	62.86	4.78	0.63	4.17	1.36
34. Stilts	95	0	4.28	1.26	3.53	1.62
35. Dominoes	90.9	72.38	4.73	0.75	4.6	1.01
36. Cactus	100	92.38	4.79	0.58	4.58	1.02
37. Escalator	99.1	92.38	4.82	0.62	4.84	0.65
38. Harp	97.3	1.9	4.76	0.67	3.87	1.53
39. Hammock	94.1	11.43	4.67	0.74	4.11	1.37
40. Knocker	97.7	21.9	4.84	0.53	3.91	1.51
41. Pelican	92.7	8.57	4.7	0.72	3.7	1.49
42. Stethoscope	95	84.76	4.74	0.61	4.65	0.95
43. Pyramid	96.8	98.1	4.67	0.72	4.64	1.03
44. Muzzle	92.7	26.67	4.67	0.76	3.44	1.67
45. Unicorn	91.3	6.67	4.74	0.64	4.13	1.43
46. Funnel	96.3	35.24	4.77	0.63	4.46	1.08
47. Accordion	81.7	2.86	4.74	0.64	3.81	1.59
48. Noose/boomerang	–	0.95	4.75	0.71	3.57	1.66
49. Asparagus	93.6	5.71	3.87	1.52	2.62	1.57
50. Compass	69	56.19	4.66	0.85	4.42	1.17
51. Latch	80	95.24	4.66	0.76	4.57	0.88
52. Tripod	89.5	13.33	4.27	1.25	3.98	1.5
53. Scroll	92.7	83.81	4.56	0.81	4.55	1.04
54. Tongs	84.5	84.76	4.82	0.46	4.74	0.73
55. Sphinx	75.8	45.71	4.66	0.76	4.3	1.29
56. Yoke	63	0	3.59	1.51	2.03	1.42
57. Trellis	77.2	0.95	3.85	1.33	2.89	1.58
58. Palette	69	34.29	4.76	0.6	4.5	1
59. Protractor	39	40.95	4.87	1.24	4.58	0.98
60. Abacus	57	18.1	4.28	1.24	4.03	1.48

The US study that we used for comparison with the pattern of errors in our sample involved the old version of the BNT, which features a noose instead of a boomerang. For our study of the KSA population, we used the second edition of the BNT-60, which features the boomerang rather than the noose (Item #48) [4,15-16]. As shown in Table 4, the participants from the KSA made a significantly low performance in identifying the drawing of a boomerang, with only one participant identifying the item correctly in Arabic (the name is shown in Table 2) and three participants responding with the English name (i.e., boomerang; Table 1).

The Arabic name for Item #48, boomerang, is خشبة مرتدة, and the participant’s answer was close and relatively correct, so credit was given for this response, as shown in Table 2, while, as mentioned, no credit was given for the English responses.

The BNT-60 FAM for our sample demonstrated a high degree of internal reliability, with an alpha value of 0.966. Item #1, bed, showed no variance, so it was deleted from the scale. The familiarity of the participants with each BNT-60 item was noted and compared with the results of another published study that evaluated the familiarity of 98 US students who were native English speakers with the BNT-60 images [17]. In our study, the average familiarity score for the participants from the KSA was 4.28, compared with 4.70 for the participants in the US study. As shown in Table 4, Item #19, pretzel, was significantly more familiar to the US participants than to the KSA participants. Specifically, in further analysis of items with a familiarity difference of > 0.5, the rating for Item #19 was 4.83 for the former and 3.92 for the latter. Similarly, the U.S. participants were more familiar with seahorse (US: 4.42; KSA: 3.90), wreath (US: 4.76; KSA: 3.64), beaver (US: 4.70; KSA: 3.74), harmonica (US: 4.38; KSA: 3.52), igloo (US: 4.78; KSA: 4.17), stilts (US: 4.28; KSA: 3.53), harp (US: 4.76; KSA: 3.87), hammock (US: 4.67; KSA: 4.11), knocker (US: 4.84; KSA: 3.91), pelican (US: 4.70; KSA: 3.70), muzzle (US: 4.67; KSA: 3.44), unicorn (US: 4.74; KSA: 4.13), accordion (US: 4.74; KSA: 3.81), asparagus (US: 3.87; SA: 2.62), tripod (US: 4.27; KSA: 3.98), yoke (US: 3.59; KSA: 2.03), and trellis (US: 3.85; SA: 2.89). When we compared the FAM rate average for the participants in our study for the item boomerang with the FAM degree of the overall average score, we found the rating for this item to be quite low, at > 0.5 (Item #48: 3.57; KSA average: 4.28).

We used Spearman’s rank to investigate the association between accurate naming and familiarity. The correlation coefficient for the relationship between NA and FAM of 0.837 (P < 0.001) showed that these assessments were closely associated. We calculated the naming agreement rates, which are shown in Table 2, as the percentage for each name given by the participants. Alternative correct responses that were consistently given by at least 10% of participants were permitted, provided that the names accurately described the item or were listed in Arabic dictionaries as synonyms for the names.

## Discussion

The BNT is a widely used neuropsychological assessment tool for measuring an individual's ability to name objects. It is widely used in the assessment and diagnosis of several neurological disorders, including traumatic brain injury, dementia, and aphasia. However, it is crucial to generate adequate normative data for different demographics and take language and cultural aspects into account to ensure an accurate interpretation of BNT results.

Numerous research studies have attempted to produce normative data for the BNT among different populations, highlighting the importance of having normative data for specific languages. For example, a study focused on the adult Spanish-speaking population in Latin America provided normative data and developed a standard version of the BNT [1]. Similarly, studies conducted on the elderly Turkish and Dominican populations produced their own BNT norms [5,10].

Furthermore, linguistic and cultural variables can significantly impact BNT performance. Barker-Collo drew attention to the potential cultural bias of the BNT and discussed possible adaptations [9].

In our study, the participants were native Arabic speakers from the KSA and highly educated university students. They performed poorly on the BNT-60 in terms of naming accuracy (NA) and overall scores. They were less familiar with many of the test items compared to the participants in the US study, with whom we compared their scores. The items "asparagus," "yoke," and "trellis" had the lowest overall scores and familiarity ratings among the KSA participants, in contrast to the US participants' average responses. This suggests that the US participants were more familiar with those items and performed better on the test. The KSA participants struggled with recognizing items related to musical instruments beause of a lack of exposure. While they recognized these items as belonging to the category of musical instruments based on media exposure, they were unfamiliar with the specific names of the instruments. The responses for the items "stilts", "hammock", "abacus", and "boomerang" were similar among the participants (Table 2).

Comparing the responses of the two participant groups to the drawings of animals (e.g., camel, beaver, and pelican), we found a significant difference in both naming accuracy and familiarity ratings. Camels are common in the KSA, while beavers and pelicans are not. The KSA participants also misrecognized the items "pretzel," "beaver," "acorn," and "pelican" as "snake," "rat," "hazelnut," and "stork," respectively. Additionally, most of them identified the wreath as either a headband or flowers, indicating their inability to recognize an object that is uncommon in the KSA population. These differences in familiarity ratings for BNT items accurately reflect the cultural differences between the Arab and US populations.

It is important to acknowledge the limitations of our research. Firstly, as our study was confined to Jeddah, the applicability of the data to other Arab and Gulf regions may vary. Further research is needed to evaluate the performance and suitability of the BNT-60 in different cultural and geographical contexts.

Secondly, our research was restricted to a subset of highly educated university students within a specific age range. This sample may not accurately reflect the age distribution and educational attainment of the general population. It is recommended that future research endeavors incorporate participants from a range of ages and educational levels to achieve a more comprehensive understanding of the BNT-60's performance among the Arabic-speaking community.

Finally, our study focused on assessing the effectiveness of the current BNT-60 items in the Arabic-speaking community, and it did not intend to produce new test items. Therefore, subsequent research could include additional objects.

## Conclusions

The primary aim of the present study was to evaluate the appropriateness of the BNT-60 for Arab populations and assess the need for a new version for this population. By comparing the mean scores of college students recruited from the city of Jeddah in the KSA with the results for the same age group from the second version of the BNT-60, we demonstrated that a significantly different version is needed for the population represented in the study. Our secondary aim was to identify items that need to be replaced. To do so, we calculated the difficulty index and compared our findings with the findings in published papers on the US population.

Moreover, the findings presented here comparing the familiarity of the items on the BNT-60 to the KSA population with their familiarity with the US population show a correlation between FAM and NA. The KSA participants, as predicted, were less familiar with many items than the participants in the US study. The findings show significant positive agreement regarding the differences in the performance of the participants in the US and KSA studies. Future studies could further explore the possibilities of substituting more familiar objects for those less familiar to the Arab population.
